# CT Manifestations of Novel Coronavirus Pneumonia: A Case Report

**DOI:** 10.4274/balkanmedj.galenos.2020.2020.2.15

**Published:** 2020-04-10

**Authors:** Peng An, Ping Song, Kai Lian, Yong Wang

**Affiliations:** 1 Department of Radiology, Xiangyang First People’s Hospital Affiliated to Hubei Medical College, Hubei, China; 2 Department of Infectious Disease and Respiratory and Critical Care, Xiangyang First People’s Hospital Affiliated to Hubei Medical College, Hubei, China; # An and Song contributed equally to this work

**Keywords:** Computed Tomography, diagnosis, influenza, novel coronavirus pneumonia, pulmonary complications

## Abstract

**Background:**

Since December 2019, the outbreak of the novel coronavirus has impacted nearly >90,000 people in more than 75 countries. In this case report, we aim to define the chest computed tomography findings of 2019-novel coronavirus associated with pneumonia and its successful resolution after treatment.

**Case Report:**

A fifty-year-old female patient, who is a businesswoman, presented with chief complaints of “fever for one week, diarrhea, anorexia, and asthenia.” Initially, she was given Tamiflu. The influenza A virus serology was negative. Three days later, levofloxacin was started because the patient’s symptoms did not improve. The novel coronavirus nucleic acid test was negative. It was noted that before the onset of the disease, the patient went to Wuhan on a business trip. Despite the given treatment, her body temperature rose to 39.2°C and she was referred to our clinic for further evaluation. Then, chest computed tomography was performed and showed bilateral multifocal ground glass opacities with consolidation which suggested viral pneumonia as a differential diagnosis, and the subsequent 2019-novel coronavirus pneumonia nucleic acid test was positive.

**Conclusion:**

Chest computed tomography offers fast and convenient evaluation of patients with suspected 2019-novel coronavirus pneumonia.

Since December 2019, 27 cases of pneumonia associated with novel coronaviruses (2019-nCoV) infection have been found in Wuhan City, Capital Of Central China’s Hubei Province. By 17:33 on February 27, 2020, the National Health Commission has received a report stating a total of 78959 confirmed cases, 2308 suspected cases, 7952 severe respiratory cases, 36157 cured cases, and 2791 cases who died because of pneumonia associated with 2019-nCoV infection in China. We report here the imaging findings of a patient who has pneumonia associated with 2019-nCoV infection in order to improve the level of confidence in diagnosis and differential diagnosis of the 2019-nCoV and to assist early detection, early report, early isolation, early diagnosis, and early treatment.

## CASE PRESENTATION

**Figure 1:**
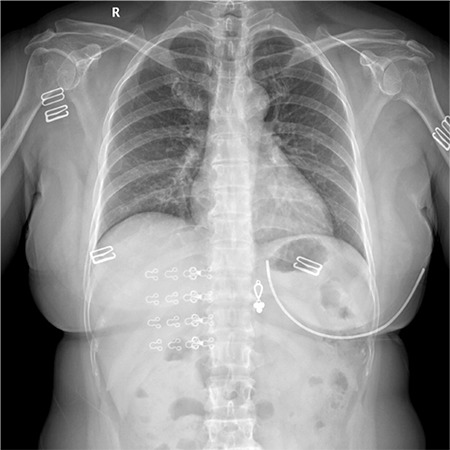
Initial admission chest X-ray shows increased and thickened right lower lung markings, suggesting bronchitis and interstitial pneumonia.

**Figure 2:**
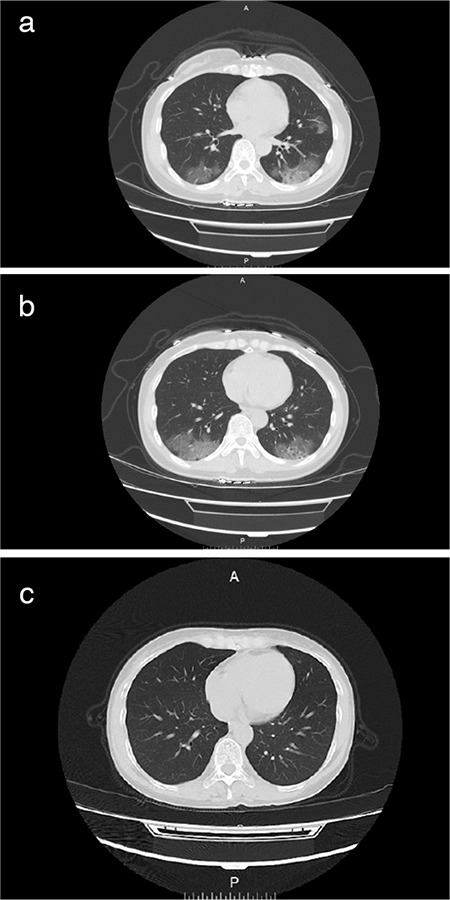
**a., b.** CT images reveal multifocal ground glass opacities with consolidation related with 2019 novel coronavirus (2019-nCoV). c. The infection foci of both lungs were absorbed and disappeared.

On February 4, 2020, a 50-year-old female patient, who is a businesswoman, presented with chief complaints of “fever for one week, diarrhea, anorexia, and asthenia,” and she was admitted to the Infectious Diseases Fever Clinic of Xiangyang First People’s Hospital Affiliated to Hubei Medical College. The patient had a five-day business trip in Wuhan (from January 22, 2020, to January 27, 2020). Fever initially occurred on January 28, 2020, with a body temperature of 38.5°C, with dry cough and muscle ache. On January 30, 2020, the patient went to Xiangyang First People’s Hospital of Traditional Chinese Medicine (TCM) for consultation in the respiratory department, and the laboratory test reported that the influenza A virus serology was negative. The results of blood routine examination were normal [white blood cell count (WBC): 5.1×10^9^/l; neutrophil percentage (neu%): 69.2%; lymphocyte percentage (lym%): 25.6%; lymphocyte absolute value (lym): 1.28×10^9^/l; C-reactive protein (CRP): 6.1 mg/l] and she was given Tamiflu (75 mg/time, twice per day) orally and was rehydrated. Three days later (on February 2, 2020), the patient still had dry cough, so she went to hospital (TCM) again. It was noted that before the onset of the disease, the patient went to Wuhan on a business trip and a novel coronavirus nucleic acid test was performed and it was negative. At admission, chest X-ray showed increased and thickened right lower lung markings, which suggested bronchitis and possible interstitial pneumonia according to her positive family history of interstitial pneumonia (Figure 1). Levofloxacin was given drip once for intravenous treatment, and Tamiflu was continued to be taken orally. On February 04, 2020, the patient's symptoms were still not improved, and her body temperature continued to rise to 39.2°C. She was referred to our fever clinic for further evaluation, and a chest computed tomography (CT) was performed. Chest CT showed bilateral multifocal ground glass opacities with consolidation which suggested viral pneumonia as a differential diagnosis (Figure 2a, 2b), and the subsequent 2019-nCoV pneumonia nucleic acid test was positive.

On February 04, 2020, the physical examination revealed persistence of acute illness status with normal physical examination findings including normal breathing sounds. Her blood lab results were as follows: WBC: 11.1×10^9^/l (↑); neu%: 90.0% (↑); neutrophil count: 9.90×10^9^/l (↑); lym%: 7.1% (↓); monocyte percentage: 2.8% (↓); eosinophil percentage: 0.1% (↓), lym: 0.77×10^9^/l (↓); CRP: 15.1 mg/l (↑).

During the course of treatment, the patient had no respiratory distress symptoms except for persistent fever and cough. Based on the novel coronavirus pneumonia diagnosis and treatment plan in China (trial version fifth), alpha interferon aerosol inhalation (dose for adults: five million U; add sterile water for injection, 2 mL, twice daily) with oral abidol (200 mg for adults, three times a day) was used in this patient. Five days after the initiation of the treatment (February 10, 2020), the patient has recovered completely, the nucleic acid retest was negative, and chest CT findings resolved (Figure 2c). After 14 days of isolation in the hotel designated by the hospital, the patient was discharged. The follow-up results (as of February 25, 2020) showed no abnormality, and the nucleic acid retesting was still negative. Written informed consent was obtained from the patient.

## DISCUSSION

Most of the early admitted patients who have pneumonia associated with 2019-nCoV infection have a history of exposure to the South China seafood market in Wuhan. According to the epidemiological investigation of these patients, it has been confirmed that the virus has the characteristics of human-to-human transmission and strong infectivity. The transmission through respiratory droplets is the main route of transmission, and it can also be transmitted by close touch. The incubation period is generally three to seven days, and the longest period is not more than 14 days. However, there were two patients who had a 20-day incubation period, but the odds were rare and minimal. People who are generally susceptible are the elderly with existing comorbidities, and additionally children and infants can also be infected (1).

Clinical manifestations of 2019-nCoV infection include fever, asthenia, and dry cough. Few patients can have symptoms such as nasal obstruction, runny nose, and diarrhea. In severe cases, dyspnea usually occurs one week later, while, in critical patients, acute respiratory distress syndrome, septic shock, metabolic acidosis, and coagulation dysfunction can occur. In some patients, the onset symptoms are mild, and no fever was present. Most patients have a good prognosis, and a few patients are critically ill or die, most of whom are elderly people having comorbidities. In the laboratory examination, in the early stage of the disease, the total number of peripheral blood leukocytes is increased or decreased, and the lymphocyte count is decreased. Some patients may have increased liver enzyme, muscle enzyme, and CRP. The novel coronavirus nucleic acid can be detected in throat swabs, sputum, lower respiratory tract secretions, and blood samples (2,3).

The CT manifestations of 2019-nCoV pneumonia are similar to that of SARS, which are usually fast change, multiple, and migratory, but the distribution of lesions is mainly in the middle and peripheral zones of the lung. At the beginning, the symptoms of the patients were mild, the total number of leukocytes and neutrophils were increased, and the antiallergic and anti-inflammatory treatments had a certain effect (1,2). Bronchopneumonia is more common in infants and elderly patients with weak bodies. Its clinical manifestations are more severe, including high fever, cough, expectoration, dyspnea, cyanosis, and chest pain. On the images, the lung texture is increased and thickened, and multiple patchy dense shadows were found in the middle and lower lung fields of both lungs, with uneven density and fuzzy margin. Dense lesions could be fused into flakes, and nodules of different sizes could be seen (3). The main manifestations of interstitial pneumonia are thickening and blurring of lung texture and interweaving into a network, accompanied by ground glass shadow, inflammatory infiltration around the hilum, increased density of hilum and a vague outline and unclear structure. In our patient who had pneumonia associated with 2019-nCoV infection, ground glass opacities suggesting inflammation of lung parenchyma was the main imaging feature, and the lesions were mainly located in the peripheral zones of both lungs. There was no abnormality in the lung hilum, but interstitial changes could occur in the later stage (4,5). Recently, chest CT has been documented to be more sensitive in detection of 2019-nCoV infection in a series of 1014 cases (6).

Chest CT offers a fast and convenient evaluation of patients with suspected pneumonia associated with 2019-nCoV and is currently included in the clinical diagnosis basis of the National Health Committee's 2019 nCoV pneumonia diagnosis and treatment plan (trial version fifth).
